# Conformational features and ionization states of Lys side chains in a protein studied using the stereo-array isotope labeling (SAIL) method

**DOI:** 10.5194/mr-2-223-2021

**Published:** 2021-04-26

**Authors:** Mitsuhiro Takeda, Yohei Miyanoiri, Tsutomu Terauchi, Masatsune Kainosho

**Affiliations:** 1 Structural Biology Research Center, Graduate School of Science, Nagoya University, Furo-cho, Chikusa-ku, Nagoya, 464-8602, Japan; 2 Department of Structural BioImaging, Faculty of Life Sciences, Kumamoto University, 5-1, Oe-honmachi, Chuo-ku, Kumamoto, 862-0973, Japan; 3 Research Center for State-of-the-Art Functional Protein Analysis, Institute for Protein Research, Osaka University, 3-2 Yamadaoka, Suita, Osaka, 565-0871, Japan; 4 SAIL Technologies Co., Inc., 2008-2 Wada, Tama-city, Tokyo, 206-0001, Japan; 5 Graduate School of Science, Tokyo Metropolitan University, 1-1 Minami-ohsawa, Hachioji, Tokyo, 192-0397, Japan

## Abstract

Although both the *hydrophobic* aliphatic chain and *hydrophilic* ζ -amino group of the Lys side chain presumably contribute to the structures and functions of proteins, the *dual* nature of the Lys residue has not been fully investigated using NMR spectroscopy, due to the lack of appropriate methods to acquire comprehensive information on its long consecutive methylene chain. We describe herein a robust strategy to address the current situation, using various isotope-aided NMR technologies. The feasibility of our approach is demonstrated for the Δ+ PHS/V66K variant of staphylococcal nuclease (SNase), which contains 21 Lys residues, including the engineered Lys-66 with an unusually low p $K_{a}$ of ∼ 5.6. All of the NMR signals for the 21 Lys residues were sequentially and stereospecifically assigned using the stereo-array isotope-labeled Lys (SAIL-Lys), [U- ${}^{13}$ C, ${}^{15}$ N; $\beta_{2}$ , $\gamma_{2}$ , $\delta_{2}$ , $\varepsilon_{3}$ -D ${}_{4}$ ]-Lys. The complete set of assigned ${}^{1}$ H, ${}^{13}$ C, and ${}^{15}$ N NMR signals for the Lys side-chain moieties affords useful structural information. For example, the set includes the characteristic chemical shifts for the ${}^{13}$ C ${}^{\delta }$ , ${}^{13}$ C ${}^{\varepsilon }$ , and ${}^{15}$ N ${}^{\zeta }$ signals for Lys-66, which has the deprotonated ζ -amino group, and the large upfield shifts for the ${}^{1}$ H and ${}^{13}$ C signals for the Lys-9, Lys-28, Lys-84, Lys-110, and Lys-133 side chains, which are indicative of nearby aromatic rings. The ${}^{13}$ C ${}^{\varepsilon }$ and ${}^{15}$ N ${}^{\zeta }$ chemical shifts of the SNase variant selectively labeled with either [ ε - ${}^{13}$ C; ε , ε -D ${}_{2}$ ]-Lys or SAIL-Lys, dissolved in H ${}_{2}$ O and D ${}_{2}$ O, showed that the deuterium-induced shifts for Lys-66 were substantially different from those of the other 20 Lys residues. Namely, the deuterium-induced shifts of the ${}^{13}$ C ${}^{\varepsilon }$ and ${}^{15}$ N ${}^{\zeta }$ signals depend on the ionization states of the ζ -amino group, i.e., - 0.32 ppm for $\Delta \delta^{13}$ C ${}^{\varepsilon }$ [N ${}^{\zeta }$ D ${}_{3}^{+}$ -N ${}^{\zeta }$ H ${}_{3}^{+}$ ] vs. - 0.21 ppm for $\Delta \delta^{13}$ C ${}^{\varepsilon }$ [N ${}^{\zeta }$ D ${}_{2}$ -N ${}^{\zeta }$ H ${}_{2}$ ] and - 1.1 ppm for $\Delta \delta^{15}$ N ${}^{\zeta }$ [N ${}^{\zeta }$ D ${}_{3}^{+}$ -N ${}^{\zeta }$ H ${}_{3}^{+}$ ] vs. - 1.8 ppm for $\Delta \delta^{15}$ N ${}^{\zeta }$ [N ${}^{\zeta }$ D ${}_{2}$ -N ${}^{\zeta }$ H ${}_{2}$ ]. Since the 1D ${}^{13}$ C NMR spectrum of a protein selectively labeled with [ ε - ${}^{13}$ C; ε , ε -D ${}_{2}$ ]-Lys shows narrow ( > 2 Hz) and well-dispersed ${}^{13}$ C signals, the deuterium-induced shift difference of 0.11 ppm for the protonated and deprotonated ζ -amino groups, which corresponds to 16.5 Hz at a field strength of 14 T (150 MHz for ${}^{13}$ C), could be accurately measured. Although the isotope shift difference itself may not be absolutely decisive to distinguish the ionization state of the ζ -amino group, the ${}^{13}$ C ${}^{\delta }$ , ${}^{13}$ C ${}^{\varepsilon }$ , and ${}^{15}$ N ${}^{\zeta }$ signals for a Lys residue with a deprotonated ζ -amino group are likely to exhibit distinctive chemical shifts as compared to the *normal* residues with protonated ζ -amino groups. Therefore, the isotope shifts would provide a useful auxiliary index for identifying Lys residues with deprotonated ζ -amino groups at physiological pH levels.

## 1 Introduction

Detailed studies on the structures and dynamics of the Lys residues in a protein have been severely hampered by the difficulty in gathering comprehensive NMR information on their side-chain moieties. It is especially challenging to establish *unambiguous* stereospecific assignments for the prochiral protons in the four consecutive methylene chain, which is the longest aliphatic chain among the 20 common amino acids. Given the lack of generally applicable strategies to overcome this obstacle, only a few NMR studies have probed the structural aspects of stereospecifically assigned Lys residues. The ionization states of the Lys ζ -amino groups also provide important information, as they are often involved in specific intra- and/or intermolecular molecular recognition processes and thus play vital roles in protein functions. Therefore, the side-chain moieties of Lys residues contribute to maintaining the structure and biological functions of a protein by two elements: the *hydrophobic* methylene chain and the *hydrophilic* ζ -amino group. To investigate this *dual* nature of the Lys side chain, we have applied various isotope-aided NMR technologies, including the stereo-array isotope labeling (SAIL) method (Kainosho et al., 2006).

The Lys ζ -amino groups, which usually have p $K_{a}$ values around 10.5, are protonated (NH ${}_{3}^{+}$ ) at around neutral pH. However, certain proteins have Lys residues with deprotonated ζ -amino groups, even at neutral or acidic pH (Harris and Turner, 2002). In such cases, the p $K_{a}$ values of the Lys ζ -amino group are substantially lowered owing to its particular local environment. Since the Lys ζ -NH ${}_{2}$ groups are endowed with significantly different physical chemical properties, as compared to the ζ -NH ${}_{3}^{+}$ , they can perform specific functions, such as Schiff base formation through nucleophilic attacks on various substrates (Highbarger et al., 1996; Barbas et al., 1997). Although the ionization states of Lys ζ -amino groups in a protein have been inferred from X-ray crystallographic maps, they are subject to misinterpretation and may not always be identical to those in solution. NMR spectroscopy provides methods for determining the charge state of Lys side chains. For example, the NH ${}_{3}^{+}$ and NH ${}_{2}$ states of Lys residues in solution can be identified from cross-peak patterns in the ${}^{1}$ H– ${}^{15}$ N correlation NMR spectra, if the hydrogen exchange rates are sufficiently slow, or from the values of ${}^{15}$ N ${}^{\zeta }$ and/or ${}^{1}$ H ${}^{\zeta }$ chemical shifts (Poon et al., 2006; Iwahara et al., 2007; Takayama et al., 2008). Under physiological conditions, however, the observations of ${}^{1}$ H– ${}^{15}$ N cross peaks are often hampered due to the rapid hydrogen exchange rates of the Lys ζ -amino groups (Liepinsh et al., 1992; Liepinsh and Otting, 1996; Otting and Wüthrich, 1989; Otting et al., 1991; Segawa et al., 2008). The ionization states can also be identified by the pH titration profiles for the ${}^{13}$ C ${}^{\varepsilon }$ and ${}^{15}$ N ${}^{\zeta }$ signals of individual Lys residues (Kesvatera et al., 1996; Damblon et al., 1996; Farmer and Venters, 1996; Poon et al., 2006; Gao et al., 2006; André et al., 2007). Unfortunately, long-term experiments such as pH titrations are hampered by the stability and solubility issues of a protein over the required pH range. Therefore, straightforward and robust alternative methods to identify Lys residues with distinct ionization states for the ζ -amino groups are highly desired.

We used a variant of staphylococcal nuclease, Δ+ PHS/V66K SNase (denoted as the SNase variant hereafter), as the model protein (Stites et al., 1991). This variant was engineered to add the following three features to the wild-type SNase: (i) introduction of three stabilizing mutations, P117G, H124L, and S128A (PHS); (ii) deletion of amino acids 44–49 and introduction of two mutations, G50F and V51N ( Δ ); and (iii) substitution of Val66 with Lys (V66K). With these three modifications, the Δ+ PHS/V66K SNase variant becomes thermally stable, even with the ζ -amino group of Lys-66 entrapped within the hydrophobic cavity originally occupied by the Val-66 side chain in the wild-type SNase. As a result, the ζ -amino group of Lys-66 in the SNase variant exhibits an unusually low p $K_{a}$ value of 5.7 (García-Moreno et al., 1997; Fitch et al., 2002).

**Figure A1:**
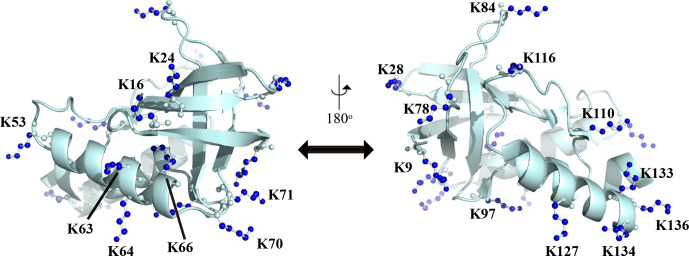
Distribution of the Lys residues in the crystalline structure of the Δ+ PHS/V66K variant of SNase (PDB#: 3HZX). All of the side-chain moieties of the Lys residues, which are shown by the ball-and-stick model in blue, exist on the protein surface, except for the Lys-66 (K66). This engineered residue is locked in the protein interior that is originally occupied by the Val side chain in the wild-type protein. Two Lys residues, K5 and K6, were not visible in the X-ray analysis of the SNase complexed with calcium and thymidine 3 ${}^{\prime }$ ,5 ${}^{\prime }$ -diphosphate, and thus it may be slightly different from that in the free state. The figure was created using PyMOL 2.4 software.

Although the SNase variant contains 21 Lys residues (Fig. A1), including the engineered Lys-66, the ${}^{13}$ C, ${}^{1}$ H, and ${}^{15}$ N NMR signals for the Lys side chains were unambiguously observed and assigned using the SNase variant selectively labeled with SAIL-Lys, i.e., L-[U- ${}^{13}$ C, ${}^{15}$ N; $\beta_{2}$ , $\gamma_{2}$ , $\delta_{2}$ , $\varepsilon_{3}$ -D ${}_{4}$ ]-Lys (Kainosho et al., 2006; Terauchi et al., 2011). In this article, we examine some of the structural features inferred from the comprehensive chemical shift data and the deuterium-induced isotope shifts on the ${}^{13}$ C ${}^{\varepsilon }$ and ${}^{15}$ N ${}^{\zeta }$ of the Lys residues in the SNase variant and show that the side-chain NMR signals can serve as powerful probes to investigate the dual nature of a Lys side chain in a protein.

## 2 Material and methods

### 2.1 Sample preparation

The Δ+ PHS/V66K SNase variants selectively labeled with either L-[U- ${}^{13}$ C, ${}^{15}$ N]-Lys, L-[U- ${}^{13}$ C, ${}^{15}$ N; $\beta_{2}$ , $\gamma_{2}$ , $\delta_{2}$ , $\varepsilon_{3}$ -D ${}_{4}$ ]-Lys (SAIL-Lys), or L-[ ε - ${}^{13}$ C; ε , ε -D ${}_{2}$ ]-Lys, which were synthesized in-house, were prepared using the *E. coli* BL21 (DE3) strain transformed with a pET3 vector (Novagen), encoding the Δ+ PHS/V66K SNase gene fused with an N-terminal His-tag. The transformed *E. coli* cells were cultured at 37 ${}^{\circ }$ C in 500 mL of M9 medium, containing anhydrous Na ${}_{2}$ HPO ${}_{4}$ (3.4 g L ${}^{-1}$ ), anhydrous KH ${}_{2}$ PO ${}_{4}$ (0.5 g L ${}^{-1}$ ), NaCl (0.25 g L ${}^{-1}$ ), D-glucose (5 g L ${}^{-1}$ ), NH ${}_{4}$ Cl (0.5 g L ${}^{-1}$ ), thiamine (0.5 mg L ${}^{-1}$ ), FeCl ${}_{3}$ (0.03 mM), MnCl ${}_{2}$ (0.05 mM), CaCl ${}_{2}$ (0.1 mM), and MgSO ${}_{4}$ (1 mM), with 10 mg L ${}^{-1}$ of the monohydrochloride salts of either [U- ${}^{13}$ C, ${}^{15}$ N]-Lys, SAIL-Lys, or [ ε - ${}^{13}$ C; ε , ε -D ${}_{2}$ ]-Lys. Each culture was maintained at 37 ${}^{\circ }$ C. An additional 20 mg L ${}^{-1}$ of each isotope-labeled Lys was supplemented when the OD ${}_{600}$ reached 0.5, and then protein expression was induced by adding isopropyl- β -D-thiogalactopyranoside (IPTG) to a final concentration of 0.4 mM. At 4–5 h after the induction, the cells were collected by centrifugation, and the SNase variant proteins were purified on a Ni-NTA column (Isom et al., 2008). The enrichment levels for Lys were ∼ 70 %, as measured using mass spectrometry. The purified proteins were dissolved in 20 mM sodium phosphate buffers containing 100 mM KCl (pH 8.0), together with a small amount of DSS as the internal chemical shift reference, prepared with either H ${}_{2}$ O, D ${}_{2}$ O, or H ${}_{2}$ O : D ${}_{2}$ O (1 : 1). The chemical shifts for ${}^{1}$ H, ${}^{13}$ C, and ${}^{15}$ N were primarily referenced to the methyl proton signal of the internal DSS according to the IUPAC recommendation (Markley et al., 1998). However, we usually convert the $\delta_{\mathrm{DSS}}$ ( ${}^{1}$ H / ${}^{13}$ C) to $\delta_{\mathrm{TSP}}$ ( ${}^{1}$ H / ${}^{13}$ C) simply by adding 0.15 ppm, i.e., $\delta_{\mathrm{DSS}}$ – $\delta_{\mathrm{TSP}}$ = 0.15 ppm, for adjustment to the previous $\delta_{\mathrm{TSP}}$ –( ${}^{1}$ H) chemical shifts reported for wt (wild-type) SNase (Torchia et al., 1989). On the other hand, all of the ${}^{15}$ N chemical shifts are referenced to DSS, to facilitate the chemical shift comparison with the recent ${}^{15}$ N data (Takayama et al., 2008). The chemical shift references are mentioned in the footnotes of the figures and tables.

### 2.2 NMR spectroscopy

The 600 MHz 2D ${}^{1}$ H– ${}^{13}$ C constant-time heteronuclear single quantum coherence correlation (HSQC) spectra of the SNase variant, selectively labeled with either [U- ${}^{13}$ C, ${}^{15}$ N]-Lys or SAIL-Lys, were measured in D ${}_{2}$ O at 30 ${}^{\circ }$ C on a Bruker Avance spectrometer equipped with a TXI cryogenic probe. For the latter sample, additional deuterium decoupling was applied during the $t_{1}$ period. The data sizes and spectral widths were 1024 ( $t_{1}$ ) × 2048 ( $t_{2}$ ) points and 12 000 Hz ( $\omega_{1}$ , ${}^{13}$ C) × 8700 Hz ( $\omega_{2}$ , ${}^{1}$ H), respectively. Each set of 32 scans per free induction decay was collected with a 1.5 s repetition time, using the ${}^{13}$ C carrier frequency at 38 ppm. The 600 MHz 3D HCCH total correlation spectroscopy (TOCSY) spectrum was measured in D ${}_{2}$ O at 30 ${}^{\circ }$ C for the SNase variant labeled with SAIL-Lys (Clore et al., 1990; Cavanagh et al., 2007). The data size and spectral width were 1024 ( $t_{1}$ ) × 32 ( $t_{2}$ ) × 2048 ( $t_{3}$ ) points and 6000 Hz ( $\omega_{1}$ , ${}^{1}$ H) Hz × 9100 Hz ( $\omega_{2}$ , ${}^{13}$ C) × 9000 Hz ( $\omega_{3}$ , ${}^{1}$ H), respectively. Each set of 16 scans/FID with a 1.5 s repetition time was collected, using the ${}^{13}$ C carrier frequency at 40 ppm.

The Lys ζ - ${}^{15}$ N signals of the SAIL-Lys-labeled SNase variant dissolved in D ${}_{2}$ O at 30 ${}^{\circ }$ C were assigned using the HECENZ pulse sequence, utilizing the out-and-back magnetization transfer from ${}^{1}$ H ${}^{\varepsilon 2}$ to ${}^{15}$ N ${}^{\zeta }$ via ${}^{13}$ C ${}^{\varepsilon }$ . The correlations between the ${}^{1}$ H ${}^{\varepsilon 2}$ and ${}^{15}$ N ${}^{\zeta }$ signals for most of the 21 Lys residues were firmly established by the pulse sequence, which was basically the same as the H2CN pulse sequence developed by André et al. (2007). The data size and the spectral width were 512 ( $t_{1}$ ) × 1024 ( $t_{2}$ ) points and 1200 Hz ( $\omega_{1}$ , ${}^{15}$ N) Hz × 9600 Hz ( $\omega_{2}$ , ${}^{1}$ H), respectively, and deuterium decoupling was applied during the $t_{1}$ period. The carrier frequencies were 38 and 28 ppm for ${}^{13}$ C and ${}^{15}$ N, respectively, and 128 scans/FID with a 2 s repetition time were accumulated.

**Figure A2:**
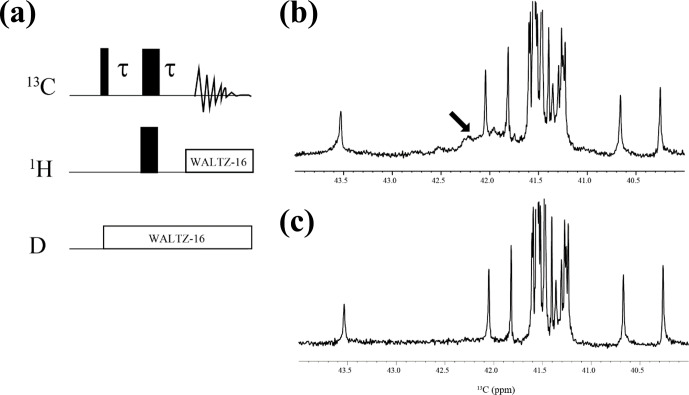
{ ${}^{1}$ H, ${}^{2}$ D}-1D ${}^{13}$ C NMR spectra for the SNase variant selectively labeled with [ ε - ${}^{13}$ C; ε , ε -D ${}_{2}$ ]-Lys. The 125.7 MHz ${}^{13}$ C NMR spectra were measured at 25 ${}^{\circ }$ C on a Bruker Avance 500 spectrometer equipped with a ${}^{13}$ C-observing DCH cryogenic probe. The broad background signals observed in the spectrum **(b)**, indicated by a thick arrow, are due to the natural abundant ${}^{13}$ C atoms bound to proton(s), which are readily filtered out to give the spectrum **(c)**, using the pulse scheme shown in panel **(a)**. The narrow and wide bars in the scheme represent 90 and 180 ${}^{\circ }$ rectangular pulses, respectively, and are applied along the x axis at τ=1.7 ms, which corresponds to 1/4 ${}^{1}$ J ${}_{\mathrm{CH}}$ . The SNase variant was dissolved in 100 % D ${}_{2}$ O buffer, containing 20 mM sodium phosphate and 100 mM potassium chloride at pH 8.0. ${}^{13}$ C chemical shifts are referenced to TSP.

The 125.7 MHz 1D ${}^{13}$ C NMR spectra of the SNase variant proteins selectively labeled with either [U- ${}^{13}$ C, ${}^{15}$ N]-Lys or [ ε - ${}^{13}$ C; ε , ε -D ${}_{2}$ ]-Lys were measured in D ${}_{2}$ O, H ${}_{2}$ O, and H ${}_{2}$ O : D ${}_{2}$ O (1 : 1), at 25 ${}^{\circ }$ C on a Bruker Avance 500 spectrometer equipped with a DCH cryogenic probe; simultaneous deuterium decoupling was achieved using the WALTZ16 scheme. The spectral width and repetition time were 6300 Hz and 5 s, respectively. In the experiment in H ${}_{2}$ O solution, a 4.1 mm o.d. Shigemi tube containing the protein solution was inserted into a 5 mm o.d. outer tube containing pure D ${}_{2}$ O for the internal lock signal. By taking advantage of the selective deuteration on the ε - ${}^{13}$ C in [ ε - ${}^{13}$ C; ε , ε -D ${}_{2}$ ]-Lys ( ∼ 98 at. %), the background ${}^{13}$ C signals due to the naturally abundant, and therefore protonated, ${}^{13}$ C nuclei were readily filtered out using the pulse scheme shown in Fig. A2.

## 3 Results and discussion

### 3.1 Complete assignment of the Lys side-chain NMR signals in the SNase variant selectively labeled with SAIL-Lys

**Figure 1:**
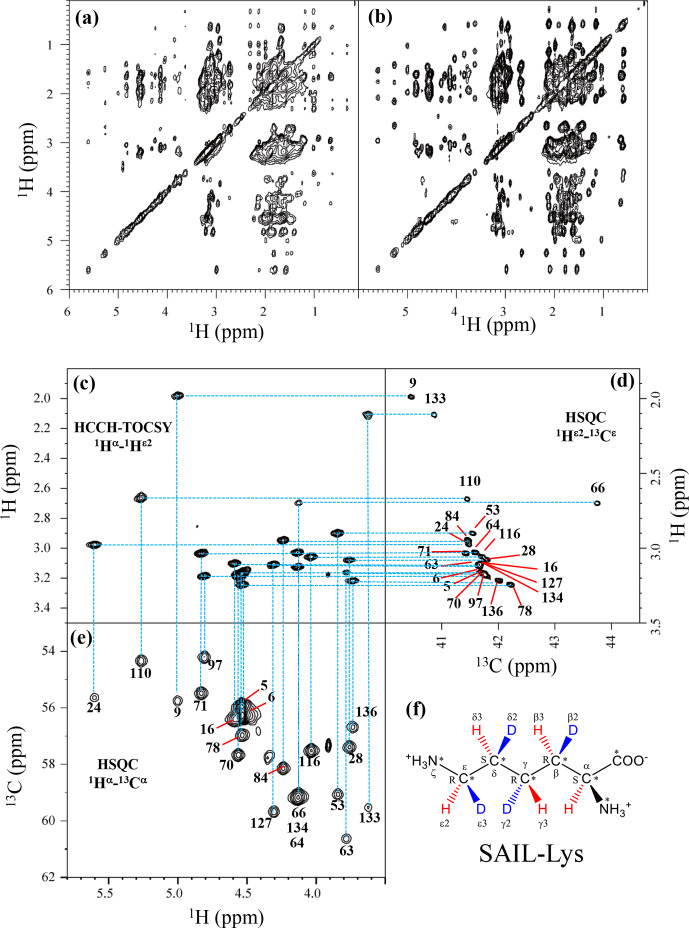
Sequential assignment of the Lys side-chain signals for the SNase variant selectively labeled with SAIL-Lys using the 3D HCCH TOCSY experiment. Panels **(a)** and **(b)** show a comparison of the F1–F3 projections of the 3D HCCH TOCSY spectra obtained for the SNase variant selectively labeled with either [U- ${}^{13}$ C, ${}^{15}$ N]-Lys **(a)** or SAIL-Lys **(b)**. A complete side-chain signal assignment was established for the SNase variant selectively labeled with SAIL-Lys by the correlation networks on the 3D HCCH TOCSY spectrum, starting from the backbone ${}^{1}$ H ${}^{\alpha }$ and ${}^{13}$ C ${}^{\alpha }$ signals with assignments deposited in the BMRB (entry #16123; Chimenti et al., 2011). For example, the ${}^{1}$ H ${}^{\varepsilon 2}$ – ${}^{13}$ C ${}^{\varepsilon }$ HSQC signals in panel **(d)** were unambiguously correlated to the backbone ${}^{1}$ H ${}^{\alpha }$ – ${}^{13}$ C ${}^{\alpha }$ HSQC signals in panel **(e)**, through the ${}^{1}$ H ${}^{\alpha }$ – ${}^{1}$ H ${}^{\varepsilon 2}$ correlation signals in panel **(c)**, which represents the F1–F3 projection of the 3D HCCH TOCSY spectrum along the ${}^{13}$ C axis (F2) restricted for the ${}^{13}$ C ${}^{\varepsilon }$ shift range of 40.1–45.5 ppm. The structure of SAIL-Lys, [U- ${}^{13}$ C, ${}^{15}$ N; $\beta_{2}$ , $\gamma_{2}$ , $\delta_{2}$ , $\varepsilon_{3}$ -D ${}_{4}$ ]-Lys, is shown in panel **(f)**. The spectrum was measured at 30 ${}^{\circ }$ C on a Bruker Avance 600 spectrometer equipped with a TXI cryogenic probe. The chemical shifts for the ${}^{1}$ H and ${}^{13}$ C dimensions are $\delta_{\mathrm{TSP}}$ .

Although the chemical shifts with sequential assignments for the backbone ${}^{1}$ H, ${}^{13}$ C, and ${}^{15}$ N signals of SNase are available in the BMRB (entry #16123; Chimenti et al., 2011), we reconfirmed them through the HNCA experiment for the [U- ${}^{13}$ C, ${}^{15}$ N]-SNase variant, since the solution conditions were slightly different. The complete side-chain assignment for all 21 Lys residues was not trivial, even for the SNase variant residue selectively labeled with [U- ${}^{13}$ C, ${}^{15}$ N]-Lys, due to the extensive signal overlap as illustrated in the F1–F3 projection of the 3D HCCH TOCSY spectrum (Fig. 1a). On the other hand, a markedly improved 3D HCCH TOCSY spectrum was obtained, under the simultaneous deuterium decoupling, for the SNase variant residue selectively labeled with SAIL-Lys (Fig. 1b), enabling us to firmly establish the full connectivity for the side-chain ${}^{1}$ H, ${}^{13}$ C, and ${}^{15}$ N NMR signals of the 21 Lys residues. To illustrate the improved spectral quality obtained with the SAIL-Lys in lieu of [U- ${}^{13}$ C, ${}^{15}$ N]-Lys, a panel obtained for the F1–F3 projection, along the ${}^{13}$ C axis (F2) restricted for the chemical shift range of 40.1–45.5 ppm for the ${}^{13}$ C ${}^{\varepsilon }$ signals, is shown for the ${}^{1}$ H ${}^{\alpha }$ – ${}^{1}$ H ${}^{\varepsilon 2}$ correlation signals (Fig. 1c). By taking advantage of the well-dispersed ${}^{1}$ H ${}^{\alpha }$ – ${}^{1}$ H ${}^{\varepsilon 2}$ signals, the backbone ${}^{1}$ H ${}^{\alpha }$ – ${}^{13}$ C ${}^{\alpha }$ signals (Fig. 1e) were readily correlated to the ${}^{1}$ H ${}^{\varepsilon 2}$ – ${}^{13}$ C ${}^{\varepsilon }$ HSQC signals (Fig. 1d). Actually, all of the SAIL-Lys side-chain ${}^{13}$ C signals were facilely and unambiguously assigned through the 3D HCCH TOCSY spectrum, yielding a complete set of the Lys side-chain NMR chemical shifts, as summarized in Table 1. It should be noted that since each one of the SAIL-Lys side-chain methylene groups (–CHD–) was *stereospecifically* deuterated, i.e., [U- ${}^{13}$ C, ${}^{15}$ N; $\beta_{2}$ , $\gamma_{2}$ , $\delta_{2}$ , $\varepsilon_{3}$ -D ${}_{4}$ ]-Lys (Fig. 1f), the Lys $\beta_{3}$ , $\gamma_{3}$ , $\delta_{3}$ , and $\varepsilon_{2}$ - ${}^{1}$ H signals of the side chains of the 21 Lys residues were stereospecifically assigned. Thus, these assigned signals have the potential of providing a wealth of information on the local conformations of the Lys side chains in solution.

**Table 1:** The ${}^{1}$ H, ${}^{13}$ C, and ${}^{15}$ N chemical shifts for the side chains of the 21 Lys residues in Δ+ PHS/V66K SNase selectively labeled with SAIL-Lys in D ${}_{2}$ O. The ${}^{1}$ H and ${}^{13}$ C signals were assigned using the 3D HCCH TOCSY experiment recorded on a Bruker 600 MHz spectrometer at 30 ${}^{\circ }$ C, pH 8.0. The ${}^{15}$ N signals were assigned using a HECENZ experiment at 600 MHz (see Fig. A4). The ${}^{1}$ H and ${}^{13}$ C chemical shifts were referenced to TSP, while the ${}^{15}$ N chemical shifts were referenced to DSS: $\delta_{\mathrm{DSS}}-\delta_{\mathrm{TSP}}$ = 0.15 ppm. Since one of the prochiral methylene protons was stereospecifically deuterated in the SAIL-Lys, i.e., [U- ${}^{13}$ C, ${}^{15}$ N; $\beta_{2}$ , $\gamma_{2}$ , $\delta_{2}$ , $\varepsilon_{3}$ -D ${}_{4}$ ]-Lys, the observed ${}^{1}$ H signals were unambiguously assigned. The chemical shifts for the engineered Lys-66, which has a deprotonated ζ -amino group, are shown in italics. The averaged chemical shifts are obtained by excluding Lys-66, and the measurement errors were estimated as less than 0.05 and 0.01 ppm, for the ${}^{13}$ C and ${}^{15}$ N and ${}^{1}$ H chemical shifts, respectively. All chemical shifts are not corrected for the isotope shifts.

	${}^{13}$ C ${}^{\alpha }$	${}^{1}$ H ${}^{\alpha }$	${}^{13}$ C ${}^{\beta }$	${}^{1}$ H ${}^{\beta 3}$	${}^{13}$ C ${}^{\gamma }$	${}^{1}$ H ${}^{\gamma 3}$	${}^{13}$ C ${}^{\delta }$	${}^{1}$ H ${}^{\delta 3}$	${}^{13}$ C ${}^{\varepsilon }$	${}^{1}$ H ${}^{\varepsilon 2}$	${}^{15}$ N ${}^{\zeta }$
K5	56.4	4.54	32.8	1.98	24.1	1.60	29.2	1.85	41.8	3.17	31.5 ${}^{a}$
K6	56.0	4.54	33.3	1.97	24.5	1.63	28.4	1.86	41.8	3.17	31.5 ${}^{a}$
K9	55.9	5.00	34.5	1.56	25.1	1.49	28.8	1.04	40.5	1.98	30.8
K16	56.6	4.60	35.6	1.92	23.9	1.47	28.3	1.78	41.7	3.10	31.7
K24	55.8	5.61	34.3	2.10	25.2	1.54	29.5	1.77	41.5	2.98	32.0
K28	57.5	3.80	29.5	2.00	24.6	0.61	29.1	1.63	41.9	3.16	31.9
K53	59.2	3.84	31.5	1.64	24.7	1.21	28.8	1.61	41.6	2.90	31.8
K63	60.8	3.78	32.8	1.91	24.2	1.46	29.7	1.75	41.8	3.17	31.5 ${}^{a}$
K64	59.4	4.13	31.8	1.89	24.5	1.55	28.9	1.74	41.6	3.03	31.6
*K66*	*59.5*	*4.12*	*32.8*	*1.85*	*25.7*	*1.76*	*34.0*	*1.47*	*43.8*	*2.70*	*20.9*
K70	57.8	4.55	32.7	1.92	24.3	1.60	28.6	1.83	41.8	3.19	31.3 ${}^{a,b}$
K71	55.6	4.84	35.2	2.01	24.4	1.60	28.4	1.82	41.4	3.04	31.7
K78	57.1	4.53	33.0	2.06	24.1	1.67	28.5	1.90	42.2	3.24	31.5
K84	58.3	4.24	31.3	1.65	23.1	0.64	28.8	1.61	41.5	2.95	32.0
K97	54.2	4.81	32.9	1.89	24.6	1.62	28.7	1.79	41.8	3.19	31.4 ${}^{a,b}$
K110	54.4	5.27	35.2	2.17	25.1	1.42	29.2	1.79	41.5	2.68	31.6
K116	57.7	4.04	31.7	1.88	24.1	1.30	28.5	1.69	41.7	3.06	31.6
K127	59.3	4.31	31.9	2.12	24.9	1.77	29.1	1.83	41.7	3.12	31.5
K133	59.6	3.62	32.2	1.42	24.1	0.59	28.9	1.15	40.9	2.10	31.7
K134	59.4	4.13	32.0	2.15	24.4	1.65	29.5	1.76	41.8	3.13	31.5
K136	56.8	3.76	28.8	1.66	24.6	1.55	28.7	1.95	42.0	3.20	31.4
Avg. δ ppm	57.4	4.40	32.7	1.90	24.4	1.40	28.9	1.71	41.6	2.98	31.6

${}^{a}$ The signals were heavily overlapped, and thus their accurate chemical shifts could not be obtained. ${}^{b}$ The ${}^{15}$ N ${}^{\zeta }$ shifts, presumably due to K70 and K97, may be reversed.

### 3.2 Structural information inferable from the Lys side-chain chemical shifts

**Figure 2:**
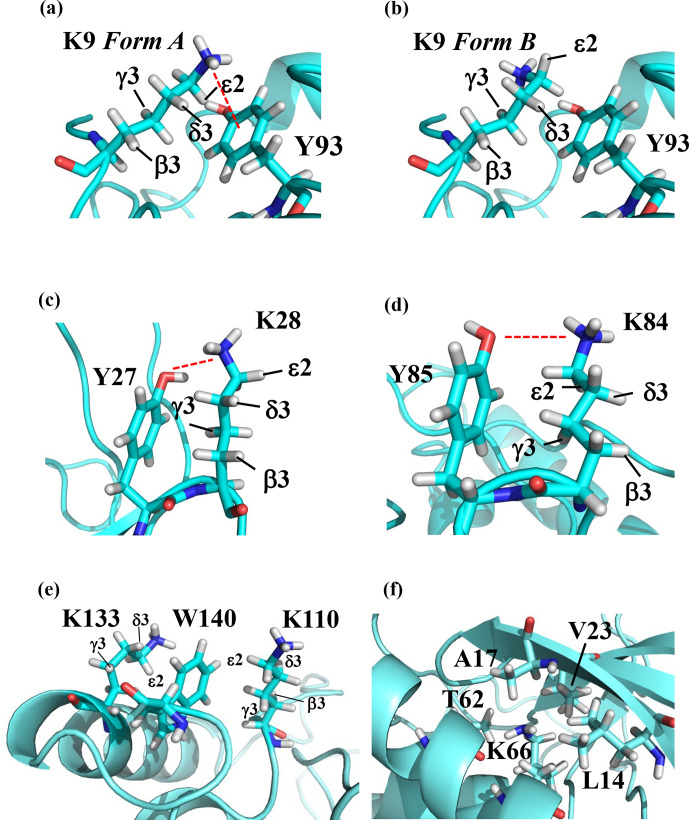
The local structures around the Lys residues, which exhibit unusual side-chain chemical shifts, in the crystal structure of the SNase variant (PDB: 3HZX). The crystal structure of the SNase variant was solved as a complex with calcium ions and thymidine 3 ${}^{\prime }$ ,5 ${}^{\prime }$ -diphosphate. Therefore, it may be slightly different from that in the free state. The figures were created with PyMOL 2.4 software in order to highlight the relative orientations between the Lys side chains and nearby aromatic rings (a)–(e) and Lys-66 and the surrounding hydrophobic amino acids (f). The atom nomenclature of the prochiral hydrogen atoms used in the figure is that of the IUPAC recommendations (Markley et al., 1998).

Note that the chemical shifts in Table 1 for the 21 Lys residues in the SAIL-Lys-labeled SNase variant are *not* corrected for the various isotope-induced shifts caused by the complicated isotope-labeling pattern of the SAIL-Lys structure (see Fig. 1f). Based on comprehensive NMR data, we should be able to elucidate the dual role of the Lys side chains in terms of the conformational dynamics and functional properties of a protein in further detail, using various solution NMR methods. In this section, we briefly interpret the chemical shift data to characterize the local conformational features by the ${}^{1}$ H, ${}^{13}$ C, and ${}^{15}$ N signals compiled in Table 1, which should be followed by more extensive studies in the future. Although we have not yet attempted to collect the comprehensive nuclear Overhauser effects (NOEs), such as using a *fully* SAIL-labeled SNase variant (Kainosho et al., 2006), it was obvious that the chemical shift data with exclusive and unambiguous assignments for the Lys residues contain an abundance of information on the side-chain conformations and ionization states of the ζ -amino groups. As described above, the unusual chemical shifts of the Lys-66 side chain confirmed the deprotonated state of its ζ -amino group. We also obtained some interesting structural information for the other Lys residues with protonated ζ -amino groups. For example, the Lys-9 side chain exists in two conformational states in the crystalline state (PDB entry #3HZX), which only differ in the $\chi^{4}$ angle, i.e., Form A (trans, ∼ - 175 ${}^{\circ }$ ) and Form B (gauche ${}^{+}$ , ∼ + 44 ${}^{\circ }$ ), as shown in Fig. 2a and b, respectively (see also Table A1). The significantly upfield-shifted signals observed for Lys-9 relative to the averaged chemical shifts ( Δδ , ppm) are obviously due to the aromatic ring current of Tyr-93, i.e., ${}^{15}$ N ${}^{\zeta }$ (30.8 ppm, Δδ=-0.8 ppm), ${}^{13}$ C ${}^{\varepsilon }{/}^{1}$ H ${}^{\varepsilon 2}$ ( 40.5/1.98 ppm, Δδ=-1.1/-1.00 ppm), and ${}^{1}$ H ${}^{\delta 3}$ (1.04 ppm, Δδ=-0.67 ppm). These chemical shifts suggest the ζ –NH ${}^{3+}$ – π interaction, as shown by the dashed red line (Fig. 2a). Therefore, the chemical shifts for Lys-9 strongly imply that the van der Waals interactions between the aliphatic side chain, as well as the *electrostatic interaction* between the positively charged ζ -HN ${}^{3+}$ and the nearby aromatic ring of Tyr-93, simultaneously contribute to preferentially stabilize the Form A conformation in solution (Fig. 2a).

**Figure A3:**
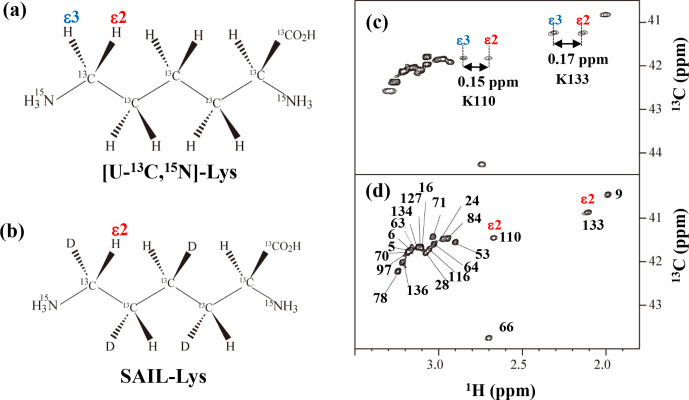
Comparison between the ${}^{13}$ C ${}^{\varepsilon }$ regions of the 2D ${}^{1}$ H– ${}^{13}$ C constant time (ct) HSQC spectra obtained for the SNase variant selectively labeled with [U- ${}^{13}$ C, ${}^{15}$ N]-Lys **(a)** and [U- ${}^{13}$ C, ${}^{15}$ N; $\beta_{2}$ , $\gamma_{2}$ , $\delta_{2}$ , $\varepsilon_{3}$ -D ${}_{4}$ ]-Lys, SAIL-Lys **(b)**. Since ε -carbons for the [U- ${}^{13}$ C, ${}^{15}$ N]-Lys residues are attached to the two prochiral protons, ${}^{1}$ H ${}^{\varepsilon 2}$ and ${}^{1}$ H ${}^{\varepsilon 3}$ , a pairwise correlation signals, namely ${}^{1}$ H ${}^{\varepsilon 2}$ – ${}^{13}$ C ${}^{\varepsilon }$ and ${}^{1}$ H ${}^{\varepsilon 3}$ – ${}^{13}$ C ${}^{\varepsilon }$ , can be observed for each of the ε -carbons **(c)**. However, considerable large chemical shift differences between the prochiral ε -methylene protons were observed only for K110 ( Δδ , 0.15 ppm) and for K133 ( Δδ , 0.17 ppm), and the other 19 Lys residues showed differences less than ∼ 0.05 ppm. On the other hand, ε -carbons for the SAIL-Lys residues are attached only to the ε 2-protons, and all of the correlation signals are automatically assigned to ${}^{1}$ H ${}^{\varepsilon 2}$ **(d)**. C ${}^{\varepsilon }$ peaks are labeled with their assignment. The spectra were measured at 30 ${}^{\circ }$ C on an Avance 600 spectrometer equipped with a TXI cryogenic probe. ${}^{1}$ H and ${}^{13}$ C chemical shifts are referenced to TSP: $\delta_{\mathrm{DSS}}$ – $\delta_{\mathrm{TSP}}$ = 0.15 ppm.

The upfield shifts of the side-chain methylenes, induced by the neighboring aromatic rings, were also detected for Lys-28, Lys-84, Lys-110, and Lys-133. Considering the local structures of Lys-28 and Lys-84 in the crystal (Fig. 2c, d), the relative orientations between Lys-28 and Tyr-27 and between Lys-84 and Tyr-85 seem to be similar to those in the crystal and are responsible for the large upfield shifts for only their ${}^{1}$ H ${}^{\gamma 3}$ signals, i.e., Lys-28: 0.61 ppm, Δδ=-0.79 ppm; Lys-84: 0.64 ppm, Δδ=-0.76 ppm, while the other ${}^{13}$ C and ${}^{1}$ H shifts remain within the average ranges (Table 1). The small but obvious low-field shifts for the ${}^{15}$ N ${}^{\zeta }$ (Lys-28: 31.9 ppm; Lys-84: 32.0 ppm; Δδ : +0.3 and 0.4 ppm, respectively) might be caused by the electrostatic interactions between the O ${}^{\eta }$ of Tyr-27 / Tyr-85 and the N ${}^{\zeta }$ of Lys-28 / Lys-84, respectively, as shown by the dashed red lines (Fig. 2c, d). The bulky indole ring of Trp-140 seems to simultaneously stabilize the aliphatic chains of both Lys-133 and Lys-110, inducing the upfield shifts for some of the side-chain signals, i.e., Lys-133 ${}^{13}$ C ${}^{\varepsilon }{/}^{1}$ H ${}^{\varepsilon 2}$ (40.9 / 2.10 ppm, Δδ=-0.7/-0.88 ppm), ${}^{1}$ H ${}^{\delta 3}$ (1.15 ppm, Δδ=-0.56 ppm), ${}^{1}$ H ${}^{\gamma 3}$ (0.59 ppm, Δδ=-0.81 ppm) and ${}^{1}$ H ${}^{\beta 3}$ (1.42 ppm, Δδ=-0.48 ppm); Lys-110 ${}^{1}$ H ${}^{\varepsilon 2}$ (2.68 ppm, Δδ=-0.30 ppm). These upfield shifted signals indicate that the van der Waals interactions between the methylene moieties of Lys-133 and Lys-110, with the hydrophobic indole ring of Trp-140 sandwiched in the middle, are also preserved in solution (Fig. 2e). Interestingly, the chemical shift differences between the two prochiral protons attached to the ε -carbons, observed for the SNase variant residue selectively labeled with [U- ${}^{13}$ C, ${}^{15}$ N]-Lys, of Lys residues 110 and 133 are considerably larger than those of the other 19 Lys residues, which are much smaller than ∼ 0.05 ppm (Fig. A3). Since the ${}^{1}$ H ${}^{\varepsilon 2}$ chemical shifts were observed at a 0.15 and 0.17 ppm higher field than the ${}^{1}$ H $\varepsilon^{3}$ chemical shifts for Lys-110 and -133, respectively, the conformations of these two Lys residues are likely to be similar to those in the crystal (Fig. 2e).

On the other hand, the unusual chemical shifts observed for the Lys-66 residue, which is trapped within the hydrophobic environment engineered in the engineered SNase variant (Fig. 2f), clearly reveal the strong influence of the ionization state of the ζ -amino group on the Lys side chain. As shown in Table 1, the ${}^{15}$ N ${}^{\zeta }$ chemical shift of the ζ -ND ${}_{2}$ of Lys-66 in the SNase variant appears at an unusually upfield position, as compared to the averaged chemical shift range for the ζ -ND ${}^{3+}$ in the other Lys residues, i.e., ${}^{15}$ N ${}^{\zeta }$ (Lys-66: 20.9 ppm, Δδ=-10.7 ppm), which is close to the value of the ζ -NH ${}_{2}$ chemical shift, 23.3 ppm, previously reported for Lys-66 in the [U- ${}^{13}$ C, ${}^{15}$ N]-SNase variant (André et al., 2007; Takayama et al., 2008). Evidently, the ${}^{15}$ N ${}^{\zeta }$ chemical shifts provide an unambiguous clue to distinguish between the deprotonated and protonated ζ -amino groups of Lys residues. However, the complete side-chain assignment including the terminal ζ - ${}^{15}$ N signals through conventional methods using a [U- ${}^{13}$ C, ${}^{15}$ N] protein is usually laborious and occasionally not practical.

In comparison with charged Lys side chains, deprotonation of the ζ -amino group of Lys-66 is characterized by sizable ${}^{1}$ H and ${}^{13}$ C chemical shift differences, i.e., ${}^{13}$ C ${}^{\varepsilon }{/}^{1}$ H ${}^{\varepsilon 2}$ ( 43.8/2.70 ppm, Δδ=+2.2/-0.28 ppm), ${}^{13}$ C ${}^{\delta }{/}^{1}$ H ${}^{\delta 3}$ ( 34.0/1.47 ppm, Δδ=+5.1/-0.24 ppm), and ${}^{13}$ C ${}^{\gamma }{/}^{1}$ H ${}^{\gamma 3}$ ( 25.7/1.76 ppm, Δδ=+1.3/+0.36 ppm). These *deprotonation* shifts, in particular, those of ${}^{13}$ C ${}^{\varepsilon }$ and/or ${}^{13}$ C ${}^{\delta }$ , could therefore be used as unambiguous indices to characterize the ionization states of the ζ -amino groups of Lys residues in a protein, since they can be accurately and readily observed and assigned using a protein selectively labeled with SAIL-Lys. It should be noted, however, that the side-chain chemical shifts in general might significantly vary according to the local environments, such as the relative position to aromatic rings, and thus the results obtained exclusively from the side-chain chemical shifts might not be absolutely reliable. To avoid any possible uncertainties in characterizing the ionization states of ζ -amino groups, an alternative approach using the deuterium-induced isotope shifts of the SAIL-Lys side-chain ${}^{13}$ C signals may be considered.

### 3.3 Characterization of the ionization state of the ζ -amino group of Lys residues using the effects of deuterium-induced isotope shifts on the side-chain ${}^{13}$ C and ${}^{15}$ N signals

In our previous studies investigating the effects of the deuterium-induced isotope shifts on the ${}^{13}$ C signals adjacent to polar functional groups with an exchangeable hydrogen, such as hydroxyl (OH) or sulfhydryl (SH) groups, we demonstrated that those isotope shifts are versatile indices for identifying residues, such as Tyr, Thr, Ser, or Cys, with *exceptionally* slow hydrogen exchange rates (Takeda et al., 2014). For example, in a protein selectively labeled with [ ζ - ${}^{13}$ C]-Tyr, the Tyr residues have much slower hydrogen exchange rates for the η -hydroxyl groups than the isotope shift differences in the ${}^{13}$ C ${}^{\zeta }$ signals and exhibit well-resolved pairwise signals with nearly equal intensities in the 1D ${}^{13}$ C NMR spectrum in H ${}_{2}$ O : D ${}_{2}$ O (1 : 1) (Takeda et al., 2009). The up- and low-field counterparts of the pairwise ${}^{13}$ C ${}^{\zeta }$ signals correspond to those in D ${}_{2}$ O and H ${}_{2}$ O, respectively, and their relative intensities reflect the fractionation factors, i.e., [OD] / [OH]. Similar approaches have been developed for Ser, Thr, and Cys residues, using the ${}^{13}$ C ${}^{\beta }$ signals observed for proteins selectively labeled with [ β - ${}^{13}$ C; β , β -D ${}_{2}$ ]-Ser, [ β - ${}^{13}$ C; β -D]-Thr, and [ β - ${}^{13}$ C; β , β -D ${}_{2}$ ]-Cys, respectively (Takeda et al., 2010, 2011). Since the isolated ${}^{13}$ C ${}^{\beta }$ (D ${}_{2}$ ) or ${}^{13}$ C ${}^{\beta }$ (D) moieties in the labeled amino acids give extremely narrow signals under the deuterium decoupling, the ${}^{13}$ C NMR signals can be obtained with remarkably high sensitivities, especially with a ${}^{13}$ C direct observing cryogenic probe. Interestingly, while the fractionation factors for the Ser and Thr hydroxyl groups, i.e., [OD] / [OH], are usually close to unity, as also for the Tyr residues, those for the Cys sulfhydryl groups, i.e., [SD] / [SH], are around 0.4–0.5 (Takeda et al., 2010, 2011). The methods are especially important, since the functional groups of the residues readily identified as having exceptionally slow hydrogen exchange rates are most likely to be involved in hydrogen bonding networks and/or located in distinctive local environments.

Although the idea of estimating the hydrogen exchange rates by the deuterium-induced isotope shifts on the ${}^{13}$ C nuclei adjacent to functional groups with exchangeable hydrogens was originally exploited years ago, for the backbone amide groups in the selectively labeled proteins with [C'- ${}^{13}$ C]-amino acid(s) (Kainosho and Tsuji, 1982; Markley and Kainosho, 1993), it has not yet been applied for the Lys ζ -amino groups. Having established the complete assignment for the 21 Lys residues in the SNase variant selectively labeled with SAIL-Lys (Table 1), we next examined the deuterium-induced chemical shifts in detail for the Lys side-chain signals. In the case of Lys residues, the NMR signals of the ζ -amino ${}^{15}$ N and ε - or δ -carbon ${}^{13}$ C signals would be plausible candidates for probing the deuterium substitution effects. There have several reports on the isotope shifts of the δ - and ε - ${}^{13}$ C for the Lys residues induced by the deuteration of ζ -amino groups (Ladner et al., 1975; Led and Petersen, 1979; Hansen, 1983; Dziembowska et al., 2004; Tomlinson et al., 2009; Platzer et al., 2014). However, it seems no comprehensive studies have applied the deuterium-induced isotope shifts on ${}^{13}$ C ${}^{\varepsilon }$ signals to characterize the ionization states of Lys residues.

**Figure 3:**
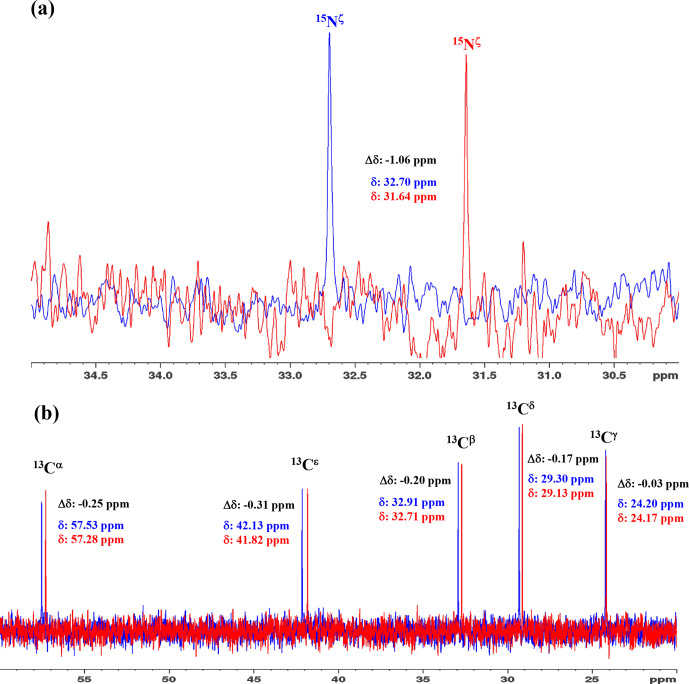
1D ${}^{15}$ N and ${}^{13}$ C NMR spectra of [ ${}^{15}$ N ${}_{2}$ ]-lysine in H ${}_{2}$ O and D ${}_{2}$ O. The 96.3 MHz 1D ${}^{15}$ N NMR spectra **(a)** and 239.0 MHz 1D ${}^{13}$ C NMR spectra **(b)** of [ ${}^{15}$ N ${}_{2}$ ]-lysine were measured at 30 ${}^{\circ }$ C on a Bruker Avance III 950 spectrometer with a TCI cryogenic probe, using ∼ 70 mM solutions of 20 mM tris buffer prepared with H ${}_{2}$ O (or D ${}_{2}$ O) at pH (or pD) 8.0. The NMR spectra and the chemical shifts, $\delta_{\mathrm{DSS}}$ ( ${}^{13}$ C / ${}^{15}$ N), shown in blue and red, are those for the H ${}_{2}$ O and D ${}_{2}$ O buffer solutions, respectively. The deuterium-induced shifts, $\Delta \delta_{\mathrm{DSS}}$ ppm : δ (in D ${}_{2}$ O) - δ (in H ${}_{2}$ O), for the ${}^{15}$ N ${}^{\zeta }$ and side-chain ${}^{13}$ C signals are shown in black.

We first examined the 1D ${}^{13}$ C and ${}^{15}$ N NMR spectra of [ ${}^{15}$ N ${}_{2}$ ]-Lys in D ${}_{2}$ O and H ${}_{2}$ O, at pH 8 and 30 ${}^{\circ }$ C, to choose the suitable NMR probes to distinguish between the deprotonated and protonated ζ -amino groups (Fig. 3). The ζ - ${}^{15}$ N signal appears at ∼ 1 ppm upfield in D ${}_{2}$ O relative to that in H ${}_{2}$ O (Fig. 3a), and the aliphatic ${}^{13}$ C signals of [ ${}^{15}$ N ${}_{2}$ ]-Lys at the natural abundance also showed isotope shifts, Δδ [ ${}^{13}$ C ${}^{i}$ (in D ${}_{2}$ O)- $\delta^{13}$ C ${}^{i}$ (in H ${}_{2}$ O)], i.e., ${}^{13}$ C ${}^{\alpha }$ , - 0.25 ppm; ${}^{13}$ C ${}^{\beta }$ , - 0.20 ppm; ${}^{13}$ C ${}^{\gamma }$ , - 0.03 ppm; ${}^{13}$ C ${}^{\delta }$ , - 0.17 ppm; and ${}^{13}$ C ${}^{\varepsilon }$ , - 0.31 ppm (Fig. 3b). Although the isotope shifts for ${}^{13}$ C ${}^{\alpha }$ and ${}^{13}$ C ${}^{\beta }$ are due to the deuteration of the α -amino group, those for ${}^{13}$ C ${}^{\delta }$ and ${}^{13}$ C ${}^{\varepsilon }$ are obviously due to the deuteration of the ζ -amino group. Considering the finding that the ${}^{13}$ C ${}^{\varepsilon }$ of Lys gives an isolated signal far from the others and exhibits a ∼ 1.8-fold larger isotope shift as compared to ${}^{13}$ C ${}^{\delta }$ , the ${}^{13}$ C ${}^{\varepsilon }$ and ${}^{15}$ N ${}^{\zeta }$ signals seem to be good candidates for probing the ionization states of Lys residues in the SNase variant.

**Table 2:** Deuterium-induced isotope shifts for the side-chain ${}^{15}$ N ${}^{\zeta }$ and ${}^{13}$ C ${}^{\varepsilon }$ signals of the 21 Lys residues in Δ+ PHS/V66K SNase. The ${}^{15}$ N ${}^{\zeta }$ chemical shift (referenced to DSS) and ${}^{13}$ C ${}^{\varepsilon }$ chemical shift (referenced to TSP) data in H ${}_{2}$ O and D ${}_{2}$ O were obtained for the SNase labeled with either SAIL-Lys or [ ε - ${}^{13}$ C; ε , ε -D ${}_{2}$ ]-Lys, respectively. Note that in the 1D ${}^{13}$ C ${}^{\varepsilon }$ data measured at 125.7 MHz, the 1D ${}^{13}$ C spectra have much higher precision as compared to those obtained using the 2D HSQC using the SNase labeled with SAIL-Lys. Spectra were recorded on a Bruker 600 MHz spectrometer at 30 ${}^{\circ }$ C, pH 8.0. The averaged chemical ${}^{13}$ C and ${}^{15}$ N chemical shifts in the last row were obtained for the Lys residues with protonated ζ -amino groups, except for Lys-66 (italics) which has a deprotonated ζ -amino group. Although the exact chemical shifts could not be obtained for the residues shown by “–”, the deuterium-induced ${}^{15}$ N and ${}^{13}$ C shifts were almost the same as those of the other residues, except for Lys-66. The averaged Δδ values show the differences between the averaged ${}^{15}$ N ${}^{\zeta }$ and ${}^{13}$ C ${}^{\varepsilon }$ , except for Lys-66, which are the differences between the ${}^{15}$ N ${}^{\zeta }$ and ${}^{13}$ C ${}^{\varepsilon }$ shifts in H ${}_{2}$ O and D ${}_{2}$ O. Negative Δδ values indicate the chemical shifts in D ${}_{2}$ O are upfield shifted due to the deuteration of the ζ -amino groups.

	$\delta^{15}$ N ${}^{\zeta }$	$\delta^{15}$ N ${}^{\zeta }$	$\Delta \delta^{15}$ N ${}^{\zeta }$	$\delta^{13}$ C ${}^{\varepsilon }$	$\delta^{13}$ C ${}^{\varepsilon }$	$\Delta \delta^{13}$ C ${}^{\varepsilon }$
	in H ${}_{2}$ O	in D ${}_{2}$ O	ppm	in H ${}_{2}$ O	in D ${}_{2}$ O	ppm
K5	–	–	–	41.89	41.54	- 0.35
K6	–	–	–	41.87	41.53	- 0.34
K9	31.9	30.8	- 1.1	40.55	40.26	- 0.29
K16	32.9	31.8	- 1.1	41.80	41.47	- 0.33
K24	33.1	32.0	- 1.1	41.66	41.31	- 0.35
K28	33.0	31.9	- 1.1	41.92	41.62	- 0.30
K53	32.9	31.8	- 1.1	–	41.36	–
K63	–	–	–	41.80	41.47	- 0.33
K64	32.7	31.6	- 1.1	41.72	41.41	- 0.31
*K66*	*22.7*	*20.9*	*–1.8*	*43.75*	*43.54*	*–0.21*
K70	–	–	–	41.89	41.55	- 0.34
K71	32.8	31.7	- 1.1	41.55	41.24	- 0.31
K78	32.7	31.5	- 1.2	42.37	42.09	- 0.28
K84	33.1	32.0	- 1.1	41.64	41.36	- 0.28
K97	–	–	–	41.91	41.59	- 0.32
K110	32.8	31.6	- 1.2	41.65	41.26	- 0.39
K116	32.8	31.6	- 1.2	41.86	41.52	- 0.34
K127	32.6	31.5	- 1.1	41.80	41.50	- 0.30
K133	32.8	31.7	- 1.1	40.96	40.67	- 0.29
K134	32.6	31.5	- 1.1	41.80	41.50	- 0.30
K136	32.5	31.4	- 1.1	42.12	41.82	- 0.30
Avg. δ , Δδ ppm	32.7	31.6	- 1.1	41.72	41.40	-0.32±0.02

**Figure 4:**
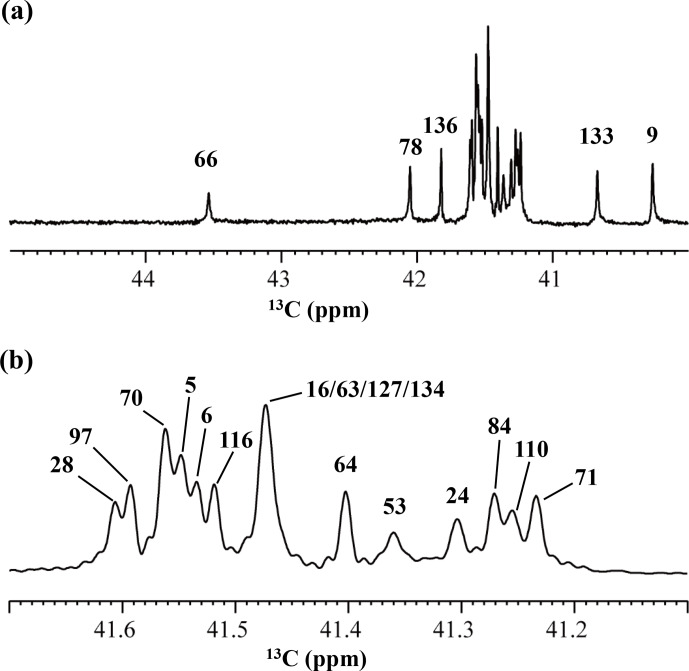
125.7 MHz { ${}^{1}$ H, ${}^{2}$ D}-decoupled 1D ${}^{13}$ C NMR spectrum for the SNase variant selectively labeled with [ ε - ${}^{13}$ C; ε , ε -D ${}_{2}$ ]-Lys. The spectra were measured at 25 ${}^{\circ }$ C, pH 8.0, in D ${}_{2}$ O solution on an Avance 500 spectrometer equipped with a DCH cryogenic probe. Although only a few discrete ${}^{13}$ C ${}^{\varepsilon }$ signals are apparent in panel **(a)**, the congested spectral region around 41–42 ppm shows well-separated signals due to their narrow line widths of 1–2 Hz **(b)**. All of the 1D NMR signals for ${}^{13}$ C ${}^{\varepsilon }$ were readily assigned using the chemical shift data, $\delta_{\mathrm{TSP}}$ ( ${}^{13}$ C) obtained from the 3D HCCH TOCSY experiment for the SNase variant selectively labeled with SAIL-Lys (see Sect. 3.1).

**Figure 5:**
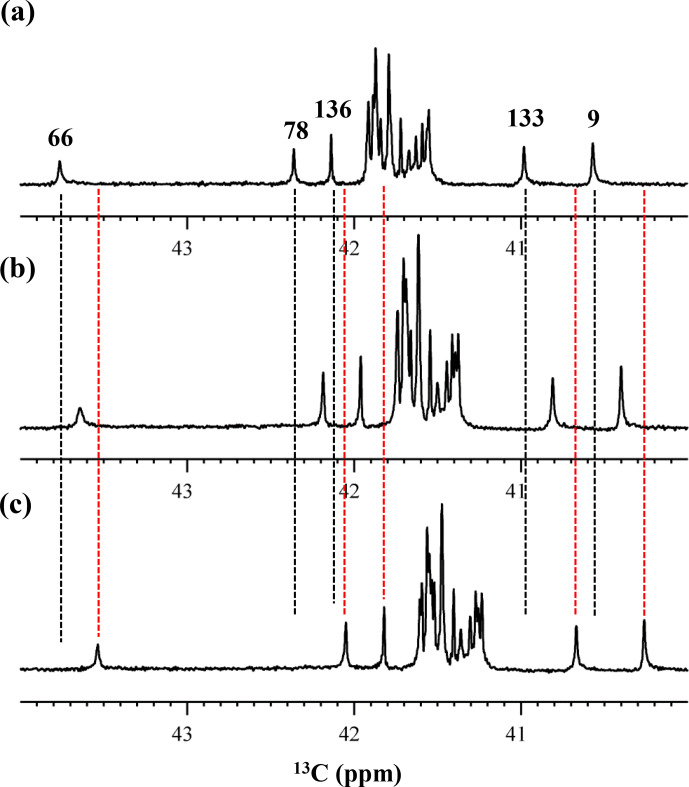
Isotope shifts on the ${}^{13}$ C ${}^{\varepsilon }$ signals of the Lys residues in the SNase variant selectively labeled with [ ε - ${}^{13}$ C; ε , ε -D ${}_{2}$ ]-Lys, caused by the deuterium substitutions for the ζ -amino groups. The 125.7 MHz { ${}^{1}$ H, ${}^{2}$ D}-decoupled 1D ${}^{13}$ C NMR spectra were measured at 25 ${}^{\circ }$ C, pH 8.0, in either H ${}_{2}$ O **(a)**, H ${}_{2}$ O : D ${}_{2}$ O (1 : 1) **(b)**, or D ${}_{2}$ O **(c)** solutions on an Avance 500 spectrometer equipped with a DCH cryogenic probe in H ${}_{2}$ O **(a)**, H ${}_{2}$ O : D ${}_{2}$ O (1 : 1) **(b)**, and D ${}_{2}$ O **(c)** solutions. The vertical dotted black and red lines show the chemical shifts observed in 100 % H ${}_{2}$ O and D ${}_{2}$ O, respectively. The complete data for the deuterium-induced isotope shifts for the side-chain ${}^{15}$ N ${}^{\zeta }$ and ${}^{13}$ C ${}^{\varepsilon }$ signals are summarized in Table 2.

Although the ${}^{15}$ N ${}^{\zeta }$ and ${}^{13}$ C ${}^{\varepsilon }$ chemical shifts for the Lys residues can be measured by the HECENZ and ${}^{1}$ H– ${}^{13}$ C ct (constant time) HSQC experiments, respectively, using the SNase variant selectively labeled with [U- ${}^{13}$ C, ${}^{15}$ N]-Lys or SAIL-Lys, it was rather difficult to determine the accurate isotope shifts of the ${}^{15}$ N ${}^{\zeta }$ and ${}^{13}$ C ${}^{\varepsilon }$ signals for all 21 Lys residues using these methods. In particular, the accurate chemical shift measurement for an individual ${}^{13}$ C ${}^{\varepsilon }$ signal was hampered by the poor quality of the ct HSQC spectrum, even for the protein labeled with SAIL-Lys (Fig. A3). Therefore, we used [ ε - ${}^{13}$ C; ε , ε -D ${}_{2}$ ]-Lys to reduce the line widths of the ${}^{13}$ C ${}^{\varepsilon }$ signals for the Lys residues in the SNase variant. As expected, the 1D ${}^{13}$ C NMR spectra of the SNase variant selectively labeled with [ ε - ${}^{13}$ C; ε , ε -D ${}_{2}$ ]-Lys showed remarkably well-resolved signals, with line widths less than 2 Hz, under the ${}^{1}$ H / ${}^{2}$ D double decoupling conditions (Fig. 4). Note that the weak background signals due to the naturally abundant ${}^{13}$ C nuclei were filtered out in this spectrum (Fig. A2). By referring to the chemical shifts in Table 1, which were determined using the 3D HCCH TOCSY experiment for the SNase labeled with SAIL-Lys, all of the 1-D ${}^{13}$ C ${}^{\varepsilon }$ signals were unambiguously assigned (Fig. 4a, b). The chemical shifts of ${}^{13}$ C ${}^{\varepsilon }$ are slightly different among the data sets, because the isotope shifts induced by the nearby isotopes on the ${}^{13}$ C ${}^{\varepsilon }$ signals are different for SAIL-Lys and [ ε - ${}^{13}$ C; ε , ε -D ${}_{2}$ ]-Lys (Tables 1, 2). The ${}^{13}$ C ${}^{\varepsilon }$ chemical shifts in H ${}_{2}$ O and D ${}_{2}$ O, which were accurately determined by the 1D ${}^{13}$ C NMR spectra, are presented in Fig. 5. At a glance, the ${}^{13}$ C ${}^{\varepsilon }$ spectra in Fig. 5a and c look almost the same, since the signals moved upfield with a constant increment of -0.32±0.02 ppm, except for the ${}^{13}$ C ${}^{\varepsilon }$ signal of Lys-66 (Table 2). Since the $\delta^{13}$ C ${}^{\varepsilon }$ values in H ${}_{2}$ O and D ${}_{2}$ O are very close to those for the *free* [ ${}^{15}$ N ${}_{2}$ ]-Lys (Fig. 3b), the ζ -amino groups are protonated in H ${}_{2}$ O and deuterated in D ${}_{2}$ O, and thus the averaged deuterium-induced isotope shift was designated as $\Delta \delta^{13}$ C ${}^{\varepsilon }$ [N ${}^{\zeta }$ D ${}_{3}^{+}$ -N ${}^{\zeta }$ H ${}_{3}^{+}$ ]. Similarly, the averaged $\Delta \delta^{15}$ N ${}^{\zeta }$ [N ${}^{\zeta }$ D ${}_{3}^{+}$ -N ${}^{\zeta }$ H ${}_{3}^{+}$ ], excluding the value for Lys-66, was determined to be -1.1±0.1 ppm (Williamson et al., 2013), which was also close to the free [ ${}^{15}$ N ${}_{2}$ ]-Lys (Fig. 3a). The $\Delta \delta^{13}$ C ${}^{\varepsilon }$ and $\Delta \delta^{15}$ N ${}^{\zeta }$ for Lys-66, which are - 0.21 and - 1.8 ppm (Table 2), respectively, confirmed that the ζ -amino group of this residue is deprotonated at pH 8 and should be designated as $\Delta \delta^{13}$ C ${}^{\varepsilon }$ [N ${}^{\zeta }$ D ${}_{2}$ -N ${}^{\zeta }$ H ${}_{2}$ ] and $\Delta \delta^{15}$ N ${}^{\zeta }$ [N ${}^{\zeta }$ D ${}_{2}$ -N ${}^{\zeta }$ H ${}_{2}$ ]. Interestingly, the fact that the averaged $\Delta \delta^{13}$ C ${}^{\varepsilon }$ [N ${}^{\zeta }$ D ${}_{3}^{+}$ -N ${}^{\zeta }$ H ${}_{3}^{+}$ ], - 0.32 ppm, was ∼ 1.5 times larger than the $\Delta \delta^{13}$ C ${}^{\varepsilon }$ [N ${}^{\zeta }$ D ${}_{2}$ -N ${}^{\zeta }$ H ${}_{2}$ ] for Lys-66, - 0.21 ppm, might suggest that the deuterium-induced isotope shift on ${}^{13}$ C ${}^{\varepsilon }$ is proportional to the number of hydrogen atoms on the ζ -amino groups. In contrast, the averaged $\Delta \delta^{15}$ N ${}^{\zeta }$ [N ${}^{\zeta }$ D ${}_{3}^{+}$ -N ${}^{\zeta }$ H ${}_{3}^{+}$ ], - 1.1 ppm, was much smaller than that of the $\Delta \delta^{15}$ N ${}^{\zeta }$ [N ${}^{\zeta }$ D ${}_{2}$ -N ${}^{\zeta }$ H ${}_{2}$ ] for Lys-66, - 1.8 ppm.

We also measured the 1D ${}^{13}$ C NMR spectrum of the SNase variant selectively labeled with [ ε - ${}^{13}$ C; ε , ε -D ${}_{2}$ ]-Lys in H ${}_{2}$ O : D ${}_{2}$ O (1 : 1), to search for the Lys residues with slowly exchanging ζ -amino groups. Clearly, there are no such residues in the SNase variant at pH 8 and 30 ${}^{\circ }$ C, as shown in Fig. 5b. Due to the rapid hydrogen exchange rates for all 21 Lys residues in this protein, the observed isotope shifts on ${}^{13}$ C ${}^{\varepsilon }$ were exactly half of the $\Delta \delta^{13}$ C ${}^{\varepsilon }$ [N ${}^{\zeta }$ D ${}_{2}$ -N ${}^{\zeta }$ H ${}_{2}$ ] for Lys-66 or $\Delta \delta^{13}$ C ${}^{\varepsilon }$ [N ${}^{\zeta }$ D ${}_{3}^{+}$ -N ${}^{\zeta }$ H ${}_{3}^{+}$ ] for the rest of the Lys residues. The hydrogen exchange rate constant ( $k_{\mathrm{ex}}$ ) for the ζ -amino group of Lys-66 in the SNase variant, which is deeply embedded in the hydrophobic cavity originally occupied by Val-66 in the wild-type SNase, was 93±5 s ${}^{-1}$ at pH 8 and - 1 ${}^{\circ }$ C (Takayama et al., 2008). Therefore, the hydrogens on the ζ -amino groups in all 21 Lys residues in the SNase variant are rapidly exchanging, and thus the observed chemical shifts for the ${}^{13}$ C ${}^{\varepsilon }$ of Lys-66 and the rest of the Lys residues in H ${}_{2}$ O : D ${}_{2}$ O (1 : 1) are the time averages for three isotopomers, NH ${}_{2}$ , NHD, and ND ${}_{2}$ , with nearly a 1 : 2 : 1 ratio for Lys-66, and for four isotopomers, NH ${}_{3}^{+}$ , NH ${}_{2}$ D ${}^{+}$ , NHD ${}_{2}^{+}$ , and ND ${}_{3}^{+}$ , with a ratio of 1 : 3 : 3 : 1. Since the time-averaged signals for Lys-66 and other Lys residues in H ${}_{2}$ O : D ${}_{2}$ O (1 : 1) appeared exactly in the middle of the spectra observed in H ${}_{2}$ O and D ${}_{2}$ O (Fig. 5a, c), the fractional factors for the isotopomers are nearly identical, as statistically random distributions.

## 4 Conclusions

In this article, we have shown that comprehensive NMR information can be obtained using cutting-edge isotope-aided NMR technologies for the Lys side-chain moieties, comprising a long hydrophobic methylene chain and a hydrophilic ζ -amino group, to facilitate hitherto unexplored investigations toward elucidating the dual nature of the Lys residues in a protein. The unambiguously assigned ${}^{13}$ C signals, together with the stereospecifically assigned prochiral protons for each of the long consecutive methylene chains, which first became available using the stereo-array isotope labeling (SAIL) method, provide unprecedented opportunities to examine the conformational features around the Lys residues in detail. The ionization states of the ζ -amino groups of Lys residues, which play crucial roles in the biological functions of proteins, could be readily characterized by the deuterium-induced isotope shifts on the ε - ${}^{13}$ C signals observed by the 1D ${}^{13}$ C NMR spectroscopy of a protein selectively labeled with [ ε - ${}^{13}$ C; ε , ε -D ${}_{2}$ ]-Lys. Both methods should work equally well for larger proteins, for which previous NMR approaches were rarely applicable. Therefore, these methods will contribute toward clarifying the structural and functional roles of the Lys residues in biologically important proteins.

## Figures and Tables

**Figure A4:**
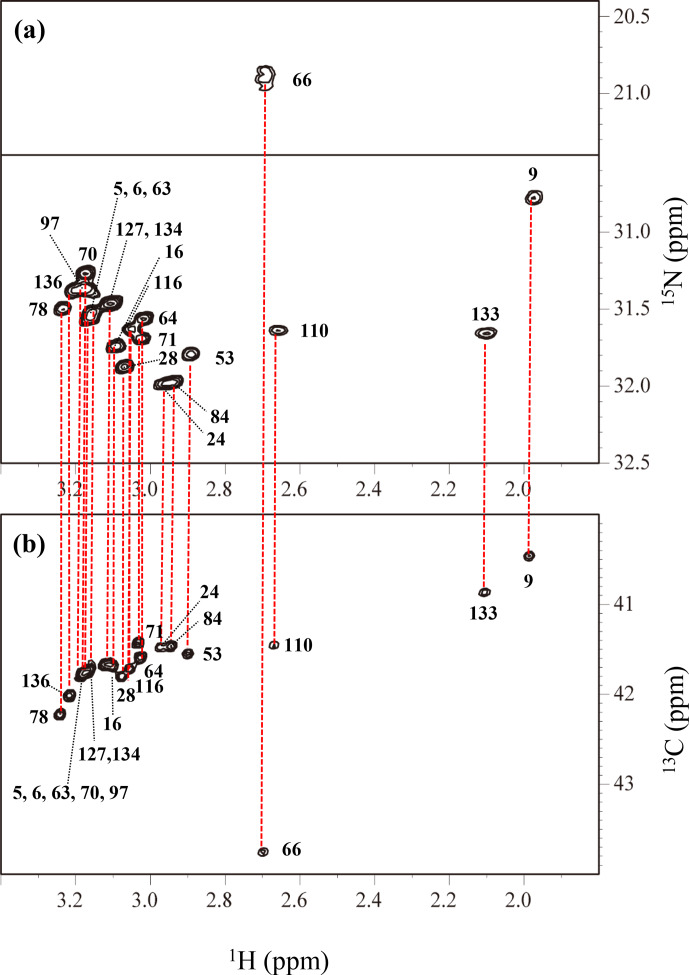
The HECENZ **(a)** and ct HSQC **(b)** spectra, recorded on a Bruker Avance 600 spectrometer ( ${}^{1}$ H, 600.0 MHz; ${}^{13}$ C, 150.9 MHz; ${}^{15}$ N, 60.8 MHz) using 1.4 mM D ${}_{2}$ O solutions of the Δ+ PHS/V66K SNase variants selectively labeled with SAIL-Lys, at 30 ${}^{\circ }$ C, pD 8.0. Note that the chemical shifts of the ${}^{1}$ H and ${}^{13}$ C dimensions are referenced to DSS, while the ${}^{15}$ N dimension is referenced to TSP: $\delta_{\mathrm{DSS}}$ – $\delta_{\mathrm{TSP}}$ = 0.15 ppm. The detailed experimental parameters are discussed in Sect. 2.2.

## Data Availability

All of the NMR data supporting this work are shown in the paper. Isotopically labeled lysines are available on request from Taiyo Nippon Sanso Co. at https://stableisotope.tn-sanso.co.jp (last access: 23 April 2021).

## Appendix A

**Table A1:** List of the dihedral angles ( $\chi^{1}$ , $\chi^{2}$ , $\chi^{3}$ , $\chi^{4}$ ) in the crystalline state (PDB #3HZX). Note: “n.d.” means “not determined”.

		$\chi^{1}$	$\chi^{2}$	$\chi^{3}$	$\chi^{4}$
K5		n.d.	n.d.	n.d.	n.d.
K6		n.d.	n.d.	n.d.	n.d.
K9	Form A	- 73	169	- 167	- 175
	Form B	- 78	171	- 153	44
K16		- 171	177	32	- 127
K24		- 170	157	146	171
K28		- 80	177	161	149
K53		- 171	164	- 164	164
K63		- 180	- 172	160	73
K64		164	179	174	166
K66		- 102	108	81	- 73
K70		82	135	171	78
K71		176	164	141	- 158
K78		- 69	- 72	- 174	- 144
K84		65	- 166	166	161
K97		175	176	- 155	- 144
K110		- 83	170	- 180	177
K116		- 148	134	99	- 165
K127		168	66	164	- 67
K133		171	156	18	- 179
K134		- 134	155	- 71	126
K136		- 68	- 112	- 143	- 50

## References

[bib1.bib1] André I, Linse S, Mulder FA (2007). Residue-specific pKa determination of lysine and arginine side chains by indirect 
${}^{15}$
N and 
${}^{13}$
C NMR spectroscopy: application to apo calmodulin. J Am Chem Soc.

[bib1.bib2] Barbas III CF, Heine A, Zhong G, Hoffmann T, Gramatikova S, Björnestedt R, List B, Anderson J, Stura EA, Wilson IA, Lerner RA (1997). Immune versus natural selection: antibody aldolases with enzymic rates but broader scope. Science.

[bib1.bib3] Cavanagh J, Fairbrother WJ, Palmer AG, Rance M, Skelton JJ (2007). Protein NMR Spectroscopy: Principles and Practice.

[bib1.bib4] Chimenti MS, Castañeda CA, Majumdar A, García-Moreno EB (2011). Structural origins of high apparent dielectric constants experienced by ionizable groups in the hydrophobic core of a protein. J Mol Biol.

[bib1.bib5] Clore GM, Bax A, Driscoll PC, Wingfield PT, Gronenborn AM (1990). Assignment of the side chain 
${}^{1}$
H and 
${}^{13}$
C resonances of interleukin-1 beta using double- and triple-resonance heteronuclear three-dimensional NMR spectroscopy. Biochemistry.

[bib1.bib6] Damblon C, Raquet X, Lian LY, Lamotte-Brasseur J, Fonze E, Charlier P, Roberts GC, Frère JM (1996). The catalytic mechanism of beta-lactamases: NMR titration of an active-site lysine residue of the TEM-1 enzyme. Proc Natl Acad Sci USA.

[bib1.bib7] Dziembowska T, Hansen PE, Rozwadowski Z (2004). Studies based on deuterium isotope effect on 
${}^{13}$
C chemical shifts. Prog Nucl Magn Reson Spectrosc.

[bib1.bib8] Farmer II BT, Venters RA (1996). Assignment of aliphatic side chain 
${}^{1}$
HN 
/
 
${}^{15}$
N resonances in perdeuterated proteins. J Biomol NMR.

[bib1.bib9] Fitch CA, Karp DA, Lee KK, Stites WE, Lattman EE, García-Moreno EB (2002). Experimental pK(a) values of buried residues: analysis with continuum methods and role of water penetration. Biophys J.

[bib1.bib10] Gao G, Prasad R, Lodwig SN, Unkefer CJ, Beard WA, Wilson SH, London RE (2006). Determination of lysine pK values using [5-
${}^{13}$
C]lysine: application to the lyase domain of DNA Pol beta. J Am Chem Soc.

[bib1.bib11] García-Moreno B, Dwyer JJ, Gittis AG, Lattman EE, Spencer DS, Stites WE (1997). Experimental measurement of the effective dielectric in the hydrophobic core of a protein. Biophys Chem.

[bib1.bib12] Hansen PE (1983). Isotope effects on nuclear shielding. Annu Rep NMR Spectrosc.

[bib1.bib13] Harris TK, Turner GJ (2002). Structural basis of perturbed pKa values of catalytic groups in enzyme active sites. IUBMB Life.

[bib1.bib14] Highbarger LA, Gerlt JA, Kenyon GL (1996). Mechanism of the reaction catalyzed by acetoacetate decarboxylase. Importance of lysine 116 in determining the pKa of active-site lysine 115. Biochemistry.

[bib1.bib15] Isom DG, Cannon BR, Castaneda CA, Robinson A, Garcia-Moreno B (2008). High tolerance for ionizable residues in the hydrophobic interior of proteins. Proc Natl Acad Sci USA.

[bib1.bib16] Iwahara J, Jung YS, Clore GM (2007). Heteronuclear NMR spectroscopy for lysine NH
${}_{3}$
 groups in proteins: unique effect of water exchange on 
${}^{15}$
N transverse relaxation. J Am Chem Soc.

[bib1.bib17] Kainosho M, Tsuji T (1982). Assignment of the three methionyl carbonyl
carbon resonances in Streptomyces subtilisin inhibitor by a carbon-13 and
nitrogen-15 double-labeling technique. A new strategy for structural studies
of proteins in solution. Biochemistry.

[bib1.bib18] Kainosho M, Torizawa T, Iwashita Y, Terauchi T, Ono AM, Güntert P (2006). Optimal isotope labelling for NMR protein structure determinations. Nature.

[bib1.bib19] Kesvatera T, Jönsson B, Thulin E, Linse S (1996). Measurement and modelling of sequence-specific pKa values of lysine residues in calbindin D9k. J Mol Biol.

[bib1.bib20] Ladner HK, Led JJ, Grant DM (1975). Deuterium isotope effects on 
${}^{13}$
C chemical shifts in amino acids and dipeptides. J Magn Reson.

[bib1.bib21] Led JJ, Petersen SB (1979). Deuterium isotope effects on carbon-13 chemical shifts in selected amino acids as function of pH. J Magn Reson.

[bib1.bib22] Liepinsh E, Otting G (1996). Proton exchange rates from amino acid side chains-implications for image contrast. Magn Reson Med.

[bib1.bib23] Liepinsh E, Otting G, Wüthrich K (1992). NMR spectroscopy of hydroxyl protons in aqueous solutions of peptides and proteins. J Biomol NMR.

[bib1.bib24] Markley JL, Kainosho M (1993). NMR of Macromolecules.

[bib1.bib25] Markley JL, Bax A, Arata Y, Hilbers CW, Kaptein R, Sykes BD, Wright PE, Wüthrich K (1998). Recommendations for the presentation of NMR structures of proteins and nucleic acids. Eur J Biochem.

[bib1.bib26] Otting G, Wüthrich K (1989). Studies of protein hydration in aqueous solution by direct NMR observation of individual protein-bound water molecules. J Am Chem Soc.

[bib1.bib27] Otting G, Liepinsh E, Wüthrich K (1991). Proton exchange with internal water molecules in the protein BPTI in aqueous solution. J Am Chem Soc.

[bib1.bib28] Platzer G, Okon M, McIntosh LP (2014). pH-Dependent random coil 
${}^{1}$
H,
${}^{13}$
C, and 
${}^{15}$
N chemical shifts of the ionizable amino acids: a guide for protein pKa measurements. J Biomol NMR.

[bib1.bib29] Poon DK, Schubert M, Au J, Okon M, Withers SG, McIntosh LP (2006). Unambiguous determination of the ionization state of a glycoside hydrolase active site lysine by 
${}^{1}$
H-
${}^{15}$
N heteronuclear correlation spectroscopy. J Am Chem Soc.

[bib1.bib30] Segawa T, Kateb F, Duma L, Bodenhausen G, Pelupessy P (2008). Exchange rate constants of invisible protons in proteins determined by NMR spectroscopy. ChemBioChem.

[bib1.bib31] Stites WE, Gittis AG, Lattman EE, Shortle D (1991). In a staphylococcal nuclease mutant the side chain of a lysine replacing valine 66 is fully buried in the hydrophobic core. J Mol Biol.

[bib1.bib32] Takayama Y, Castañeda CA, Chimenti M, García-Moreno B, Iwahara J (2008). Direct evidence for deprotonation of a lysine side chain buried in the hydrophobic core of a protein. J Am Chem Soc.

[bib1.bib33] Takeda M, Jee J, Ono AM, Terauchi T, Kainosho M (2009). Hydrogen exchange rate of tyrosine hydroxyl groups in proteins as studied by the deuterium isotope effect on C
ζ
 chemical shifts. J Am Chem Soc.

[bib1.bib34] Takeda M, Jee J, Terauchi T, Kainosho M (2010). Detection of the sulfhydryl groups in proteins with slow hydrogen exchange rates and determination of their proton/deuteron fractionation factors using the deuterium-induced effects on the 
${}^{13}$
C
β
 NMR signals. J Am Chem Soc.

[bib1.bib35] Takeda M, Jee J, Ono AM, Terauchi T, Kainosho M (2011). Hydrogen exchange study on the hydroxyl groups of serine and threonine residues in proteins and structure refinement using NOE restraints with polar side chain groups. J Am Chem Soc.

[bib1.bib36] Takeda M, Miyanoiri Y, Terauchi T, Yang C-J, Kainosho M (2014). Use of H/D isotope effects to gather information about hydrogen bonding and hydrogen exchange rates. J Magn Reson.

[bib1.bib37] Terauchi T, Kamikawai T, Vinogradov MG, Starodubtseva EV, Takeda M, Kainosho M (2011). Synthesis of stereoarray isotope labeled (SAIL) lysine via the “head-to-tail” conversion of SAIL glutamic acid. Org Lett.

[bib1.bib38] Tomlinson JH, Ullah S, Hansen PE, Williamson MP (2009). Characterization of Salt bridges to Lysines in the Protein G B1 Domain. J Am Chem Soc.

[bib1.bib39] Torchia DA, Sparks SW, Bax A (1989). Staphylococcal nuclease: Sequential assignments and solution structure. Biochemistry.

[bib1.bib40] Williamson MP, Hounslow AM, Ford J, Fowler K, Hebditch M, Hansen PE (2013). Detection of salt bridges to lysines in solution in barnase. Chem Commun.

